# Identification of miR-320d as a Negative Regulator of Proliferation and Fatty Acid Synthesis via Targeting SCD in Ovine Tail Preadipocytes

**DOI:** 10.3390/ani16132071

**Published:** 2026-07-04

**Authors:** Yaling Yang, Wujun Liu, Hang Cao

**Affiliations:** Department of Animal Science, Xinjiang Agricultural University, Urumqi 830052, China; yangyaling3141@163.com (Y.Y.); lwj_ws@163.com (W.L.)

**Keywords:** *Ovis aries*, fat deposition, miR-320d, miR-151b, preadipocyte proliferation, targeted regulation

## Abstract

Excessive tail fat deposition in sheep limits feed conversion efficiency and carcass quality, making the genetic improvement of this trait a priority. This study aimed to elucidate the molecular mechanisms regulating ovine fat metabolism. We analyzed the tissue specific expression of two candidate microRNAs, miR-320d and miR-151b, alongside their target genes *SCD* and *ACACA*, across tissues from fat-tailed and thin-tailed sheep using quantitative real-time PCR. The regulatory function of the tail fat specific miR-320d was further validated in vitro using isolated ovine tail preadipocytes. Results indicated that miR-320d and miR-151b are specifically highly expressed in tail and subcutaneous adipose tissues, respectively, and correlate negatively with their target genes. Dual-luciferase reporter assays confirmed that miR-320d directly targets the 3′UTR of *SCD* and suppresses its transcription. Overexpression of miR-320d significantly inhibited the proliferation of tail preadipocytes and downregulated downstream genes in the fatty acid metabolism pathway, including *ACACA*, *ELOVL6*, *ELOVL5*, and *FASN*. In conclusion, miR-320d exerts a negative regulatory effect on sheep tail fat deposition by suppressing preadipocyte proliferation and fatty acid synthesis, while miR-151b emerges as a potential candidate for subcutaneous fat regulation.

## Simple Summary

Fat-tailed sheep store excessive energy as tail fat. While historically useful for survival, this physiological trait reduces feed efficiency in modern agriculture. This study explored the genetic mechanisms controlling fat accumulation in different sheep body parts. We examined two specific small genetic molecules naturally present in sheep. One molecule is highly active in tail fat, while the other is active in skin fat. We further tested the tail fat molecule and found it works as a brake on fat synthesis pathways by turning off a key gene. Increasing this small molecule in sheep fat cells caused them to multiply slower and reduced the activity of downstream genes related to fat production. These findings uncover natural genetic mechanisms limiting fat expansion and provide the agricultural industry with potential targets to breed new sheep varieties with smaller tails.

## 1. Introduction

*Ovis aries* (sheep), one of the earliest domesticated herbivorous livestock, possess abundant germplasm resources and excellent economic traits. Historically, as sheep migrated to arid and semi-arid regions with harsh, prolonged cold winters and frequent nutritional scarcity, they developed a unique physiological characteristic: storing excessive energy as fat in their tails [1]. This fat-tailed trait is a classic example of evolutionary adaptation to extreme climatic and environmental conditions, acting as a vital physiological buffer against starvation and cold stress. Consequently, approximately 25% of sheep breeds worldwide are classified as fat-tailed sheep [1], and the profound phenotypic and genetic diversity between fat-tailed and thin-tailed lineages makes them ideal models for exploring the complex regulatory networks of depot-specific adipose tissue expansion [2,3]. However, with the modern mutton sheep industry moving towards large-scale and intensive development, excessive tail fat deposition has become a distinct disadvantage. It leads to problems such as low feed conversion efficiency, poor carcass quality, and reduced mating convenience, which directly restrict the improvement of production efficiency [4].

The core difference in fat deposition efficiency stems from the morphological and quantitative characteristics of adipocytes, and the cellular properties of adipose tissues vary significantly across different body parts [2]. Previous studies have confirmed that compared with thin-tailed sheep, the diameter and area of tail adipocytes in fat-tailed sheep are significantly larger [5]; within the same breed, the diameter of sheep tail adipocytes is also larger than that of other adipose tissues [6,7]. Furthermore, earlier phenotypic evaluations demonstrated that the average diameters of tail fat and subcutaneous fat adipocytes in fat-tailed Bashbay sheep were significantly larger than those in thin-tailed F2 hybrids of wild argali and Bashbay sheep (*p* < 0.01) [2]. At the cellular level, the expansion of adipose tissue is mainly driven by the increase in adipocyte number and size, rendering it a complex metabolic and endocrine organ [8]. The progression from preadipocytes to mature adipocytes, known as adipogenesis, is a highly regulated sequence involving multiple transcription factors and represents a key physiological process for lipid accumulation [9].

MicroRNAs (miRNAs), acting as endogenous non-coding short RNAs, can inhibit the transcription and translation of target genes through base pairing, playing a crucial regulatory role in this adipogenic process. As early as 2003, miRNAs were proven to regulate fat synthesis in *Drosophila* [10]. Subsequently, miR-143 became the first miRNA identified in mammalian adipogenesis, and since then, an increasing number of miRNAs have been linked to lipid synthesis [11]. In sheep research, existing studies have clearly demonstrated that miRNAs are essential factors affecting lipid metabolism and preadipocyte differentiation [12,13]. For instance, integrated miRNA and messenger RNA analyses on fat-tailed Kazakh sheep and thin-tailed Tibetan sheep further revealed that core genes governing fat deposition are under tight miRNA regulation [14]. Among the core lipogenic factors, *SCD* (Stearoyl-CoA Desaturase) acts as a critical endoplasmic reticulum-localized enzyme responsible for converting saturated fatty acids into monounsaturated fatty acids, a rate-limiting step strictly required for triglyceride synthesis and lipid droplet accumulation [15]. While the role of *SCD* in ruminant fatty acid metabolism is well established, its upstream epigenetic regulation remains an active area of research. Specifically, miR-320d, a member of the highly conserved miR-320 family, has been implicated in cellular energy homeostasis in previous studies. However, its precise regulatory role and specific targets in the context of ruminant adipose tissue development, particularly regarding *SCD* modulation, require further elucidation.

Building upon previous transcriptome sequencing and bioinformatics analyses of tail fat tissues from thin-tailed and fat-tailed sheep, a comprehensive lncRNA-miRNA-mRNA network was constructed, identifying specific miRNAs and their potential target genes related to sheep tail fat deposition [2,3,16]. Based on these foundational transcriptomic data, the present study systematically analyzed the tissue specific expression of two candidate miRNAs (miR-320d and miR-151b) alongside their predicted target genes (*SCD* and *ACACA*) [16]. To address the complex regulatory network of sheep tail fat deposition, we adopted a hierarchical validation strategy. Focusing primarily on miR-320d, which exhibits specific high expression in tail fat tissue, we conducted deep functional verification to clarify its targeted regulatory mechanisms in sheep tail fat preadipocyte proliferation and lipid metabolism. Concurrently, we sought to preliminarily outline the expression profile of miR-151b to explore its potential involvement in subcutaneous fat regulation. Ultimately, this study aims to provide critical molecular evidence for deciphering the metabolic differences between fat-tailed and thin-tailed sheep and offer scientific targets for breeding new sheep strains with optimized fat distribution.

## 2. Materials and Methods

### 2.1. Experimental Animals and Sample Collection

Healthy 3-year-old rams with similar body weights, raised under identical feeding and management conditions, were selected from a sheep farm in Tacheng, Xinjiang, China. The thin-tailed group (SW, *n* = 6) consisted of F2 hybrids of wild argali (*Ovis ammon*) × Bashbay sheep, and the fat-tailed group (ZW, *n* = 6) consisted of purebred Bashbay sheep. All experimental animals were slaughtered after 24 h of fasting and 2 h of water deprivation. Fourteen tissue samples were collected from each individual across both populations. These included visceral tissues (heart, liver, spleen, lung, kidney, rumen, small intestine), muscle tissues (longissimus dorsi, triceps brachii, quadriceps femoris), and adipose tissues (perirenal fat, perienteric fat, subcutaneous fat, tail fat). Tissue samples (with at least three biological replicates per tissue) were immediately placed into sterile cryopreservation tubes, snap-frozen in liquid nitrogen, and stored at −80 °C for total RNA extraction. Concurrently, additional fresh adipose tissue samples were collected in sterile PBS buffer and transported to the laboratory on ice for subsequent preadipocyte isolation and culture.

### 2.2. RNA Extraction and Quantitative Real-Time PCR (RT-qPCR)

Total RNA was extracted from the 14 tissue types and cultured cells using TRIzol^®^ LS Reagent (Ambion, Austin, TX, USA) according to the previously described Trizol reagent method [17]. RNA concentration and purity were assessed using an OD-1000+ Spectrophotometer (OneDrop, Nanjing, China). Reverse transcription was performed using the PrimeScript^™^ RT reagent Kit (TaKaRa, Kusatsu, Japan). The miRNA primers were designed using the stem-loop method (approx. 45 bp in length), and target gene primers were designed following the intron-spanning principle [18]. The U6 snRNA and β-actin genes were utilized as internal references for miRNA and target gene quantification, respectively. All primers were synthesized by Sangon Biotech Co., Ltd. (Shanghai, China). The specific primer sequences are listed in Table 1. The RT-qPCR [16] was performed using SYBR^®^ Premix Ex Taq™ II (TaKaRa, Kusatsu, Japan) on a CFX96™ Optics Module (Bio-Rad, Hercules, CA, USA). All samples were analyzed with three technical replicates, and the relative expression levels of miR-320d/*SCD* and miR-151b/*ACACA* were calculated using the 2^−ΔΔCt^ method [19].

### 2.3. Primary Culture and Adipogenic Induction of Ovine Tail Preadipocytes

Primary ovine tail preadipocytes were aseptically isolated in a biosafety cabinet from the tail fat tissues of the aforementioned 3-year-old purebred fat-tailed Bashbay sheep. Adipose tissues were washed with sterile PBS to remove impurities and blood residues, finely minced, and digested with two volumes of type II collagenase (Sigma-Aldrich, St. Louis, MO, USA) at 37 °C for 1.5 h under continuous shaking. Following complete tissue dissociation, digestion was terminated using an equal volume of complete medium. The cell suspension was filtered through a 200-mesh strainer and centrifuged at 1500 rpm for 6 min using an Eppendorf 5810R centrifuge (Eppendorf, Hamburg, Germany). The harvested preadipocytes were resuspended in DMEM-F12 complete medium (Gibco, Grand Island, NY, USA), seeded at an appropriate density, and cultured at 37 °C in a humidified incubator with 5% CO_2_. The culture medium was refreshed every two days.

To establish the adipogenic induction model, cells reaching approximately 80% confluence were cultured for an additional 48 h before being induced with an adipogenic induction medium (Day 0). The specific composition of the adipogenic induction medium was DMEM/F12 supplemented with 10% FBS, 0.5 mM IBMX (Gibco), 1 μM dexamethasone (Gibco), and 10 μg/mL insulin (Sigma-Aldrich). After 48 h of induction, the medium was replaced with a maintenance medium (DMEM/F12 containing 10% FBS and 10 μg/mL insulin) for another 48 h. Subsequently, cells were cultured in the complete medium with routine changes. Cell samples were collected at 0, 2, 4, 6, and 8 days post-induction and stored at −80 °C for subsequent RNA extraction and RT-qPCR assays.

### 2.4. Dual-Luciferase Reporter Assay

A dual-luciferase reporter assay system was employed to verify the targeted binding between miR-320d and the *SCD* gene. Recombinant luciferase plasmids containing the wild-type (WT) and mutant-type (MUT) sequences of the *SCD* 3′ untranslated region (3′UTR) were constructed. The constructed recombinant plasmids were co-transfected into 293T cells alongside miR-320d mimics or negative control (miR-320d NC). At 48 h post-transfection, luciferase activity was quantified using a GENios Pro microplate reader according to the manufacturer’s instructions for the dual-luciferase reporter assay kit (Promega, Madison, WI, USA). The relative luciferase intensity was calculated to validate the direct targeting interaction.

### 2.5. Cell Transfection and Viability Assay

To explore the regulatory role of miR-320d, three experimental groups were established: the miR-320d overexpression group (transfected with miR-320d mimics), the negative control group (transfected with miR-320d NC), and the miR-320d inhibition group (transfected with miR-320d inhibitors). All RNA oligonucleotides were synthesized by GenePharma (Shanghai, China). Ovine tail preadipocytes were seeded in 24-well plates and transfected using Lipofectamine^TM^ 3000 (Invitrogen, Carlsbad, CA, USA) when confluence reached 70–90%. Transfection efficiency was verified at 48 h post-transfection via RT-qPCR.

Following transfection, cells from the three groups were subjected to the aforementioned adipogenic induction conditions. Cell suspensions at 0, 2, 4, and 8 days post-induction were seeded into 96-well plates with three technical replicates per group. Cell viability was evaluated using a Cell Counting Kit-8 (CCK-8; Solarbio, Beijing, China). CCK-8 reagent was added to each well, and cells were incubated at 37 °C with 5% CO_2_ for 2 h. The absorbance at 450 nm was measured using a GENios Pro microplate reader to assess cell proliferative activity. For functional gene expression profiling, cells harvested on day 2 of induction were subjected to RT-qPCR to determine the expression of miR-320d, *SCD*, and downstream fatty acid metabolism genes (*ACACA*, *ELOVL6*, *ELOVL5*, and *FASN*).

### 2.6. Statistical Analysis

All data were analyzed using GraphPad Prism software 8.0 (GraphPad Software, San Diego, CA, USA). Data are presented as the mean ± standard deviation (SD, *n* = 3). Differences between two groups were analyzed using Student’s *t*-test. Comparisons among multiple groups were performed using a one-way analysis of variance (ANOVA) followed by Duncan’s post hoc test. Differences were considered statistically significant at *p* < 0.05, and extremely significant at *p* < 0.01.

## 3. Results

### 3.1. Expression Patterns of miR-320d and SCD in Tissues of Different Tail-Type Sheep

**Figure 1:**
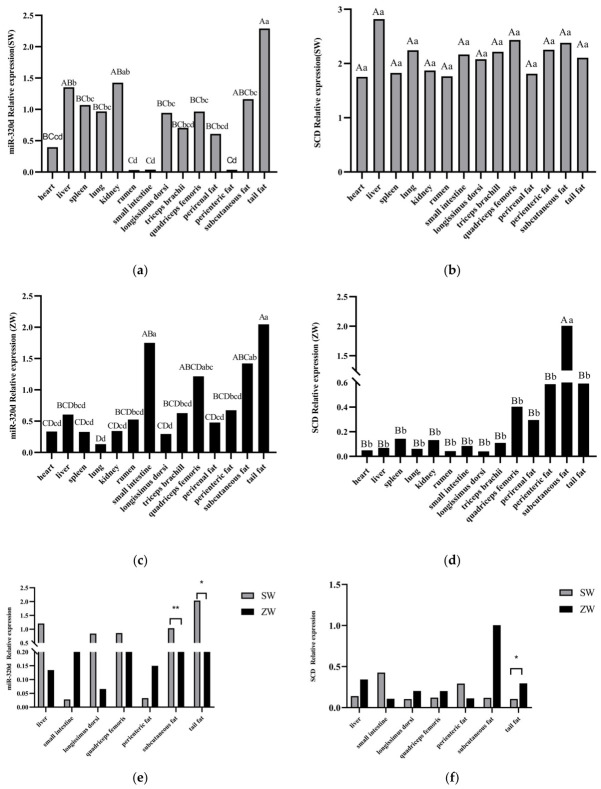
Expression levels of miR-320d and *SCD* in different tissues of two tail-type sheep. (**a**) Expression pattern of miR-320d in thin-tailed sheep tissues. (**b**) Expression pattern of *SCD* in thin-tailed sheep tissues. (**c**) Expression pattern of miR-320d in fat-tailed sheep tissues. (**d**) Expression pattern of *SCD* in fat-tailed sheep tissues. (**e**) Comparison of miR-320d expression in tail fat between thin-tailed and fat-tailed sheep. (**f**) Comparison of *SCD* expression in tail fat between thin-tailed and fat-tailed sheep. Different lowercase letters indicate significant differences at *p* < 0.05, and different uppercase letters indicate significant differences at *p* < 0.01. The absence of letters or the same letters indicates no significant difference (*p* > 0.05). * indicates a significant difference at *p* < 0.05, and ** indicates a significant difference at *p* < 0.01. SW: thin-tailed group (F2 hybrids of wild argali × Bashbay sheep); ZW: fat-tailed group (Bashbay sheep).

In thin-tailed sheep (F2 hybrids of wild argali × Bashbay sheep), miR-320d exhibited the highest expression in tail adipose tissue (Figure 1a), which was significantly higher than that in the heart, spleen, lung, rumen, small intestine, longissimus dorsi, triceps brachii, quadriceps femoris, perirenal fat, and perienteric fat (*p* < 0.01), as well as the liver and subcutaneous fat (*p* < 0.05). The target gene *SCD* peaked in the liver with no significant expression differences among the other 13 tissues (*p* > 0.05) (Figure 1b). In fat-tailed Bashbay sheep, tail adipose tissue presented the highest miR-320d expression (Figure 1c), which was significantly higher than that in the heart, liver, spleen, lung, kidney, rumen, longissimus dorsi, triceps brachii, perirenal fat, and perienteric fat (*p* < 0.01). *SCD* expression in subcutaneous fat was significantly higher than that in all other tissues (*p* < 0.01), with relatively high levels also observed in tail fat, perienteric fat, and quadriceps femoris (Figure 1d). Compared with thin-tailed sheep, fat-tailed sheep showed significantly lower miR-320d expression and higher *SCD* expression in tail fat (Figure 1e,f).

### 3.2. Expression Patterns of miR-151b and ACACA in Tissues of Different Tail-Type Sheep

**Figure 2:**
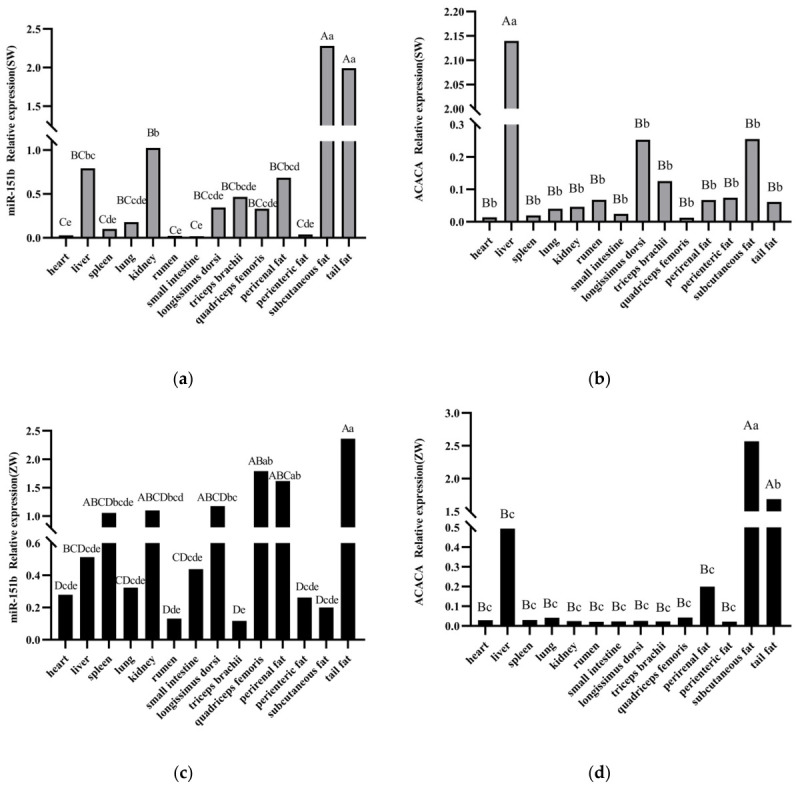

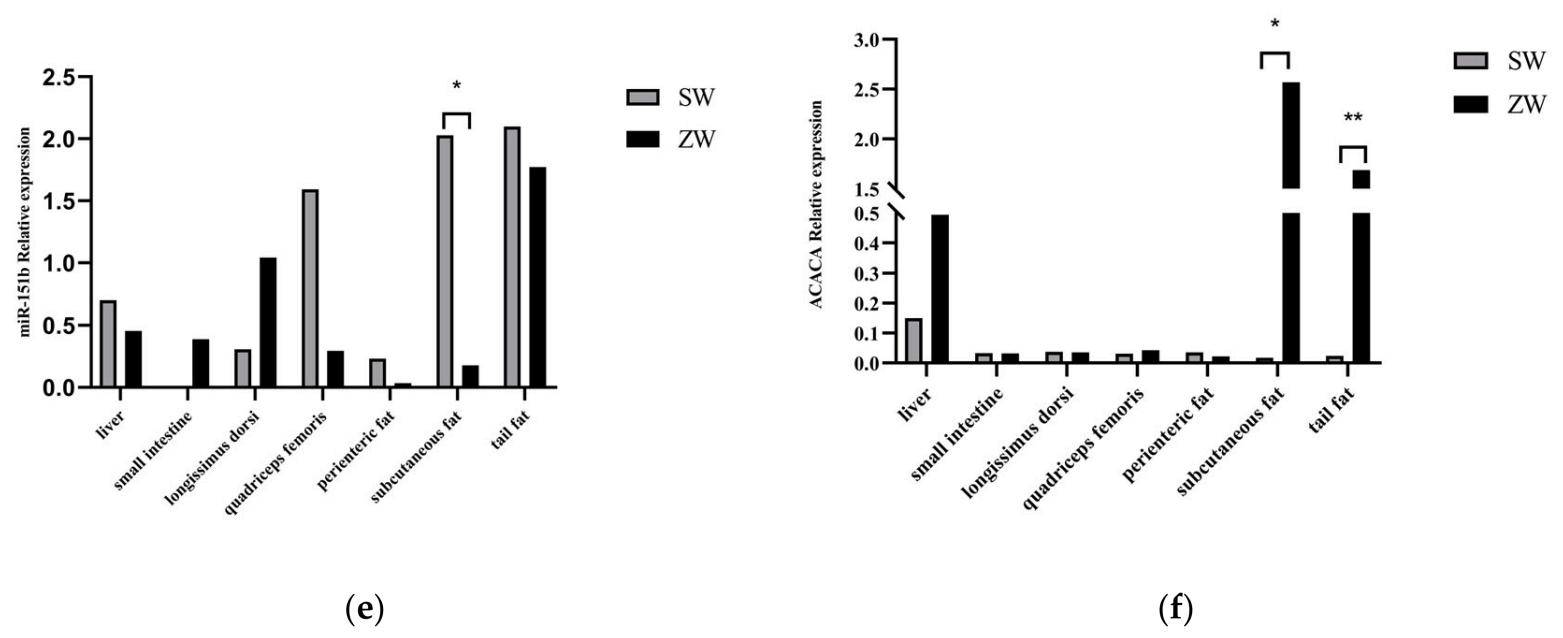
Expression levels of miR-151b and *ACACA* in different tissues of two tail-type sheep. (**a**) Expression pattern of miR-151b in thin-tailed sheep tissues. (**b**) Expression pattern of *ACACA* in thin-tailed sheep tissues. (**c**) Expression pattern of miR-151b in fat-tailed sheep tissues. (**d**) Expression pattern of *ACACA* in fat-tailed sheep tissues. (**e**) Comparison of miR-151b expression in subcutaneous fat between thin-tailed and fat-tailed sheep. (**f**) Comparison of *ACACA* expression in subcutaneous fat between thin-tailed and fat-tailed sheep. Different lowercase and uppercase letters indicate significant differences at *p* < 0.05 and *p* < 0.01, respectively. * indicates a significant difference at *p* < 0.05, and ** indicates a significant difference at *p* < 0.01. SW: thin-tailed group (F2 hybrids of wild argali × Bashbay sheep); ZW: fat-tailed group (Bashbay sheep).

In thin-tailed sheep, miR-151b was predominantly enriched in tail fat and subcutaneous fat (Figure 2a), with expression levels significantly higher than those in the other 12 tissues (*p* < 0.01). *ACACA* expression peaked in the liver, showing significant differences from other tissues (*p* < 0.05), whereas no significant differences existed among the remaining tissues (*p* > 0.05) (Figure 2b). In fat-tailed sheep, tail fat had the highest miR-151b expression (Figure 2c), which was significantly higher than that in the heart, liver, lung, rumen, small intestine, triceps brachii, perienteric fat, and subcutaneous fat (*p* < 0.01), as well as the spleen, kidney, and longissimus dorsi (*p* < 0.05). *ACACA* expression in tail fat and subcutaneous fat was significantly higher than that in other tissues (*p* < 0.01), with no significant differences among the residual tissues (*p* > 0.05) (Figure 2d). Fat-tailed sheep had significantly lower miR-151b expression in tail fat and subcutaneous fat (*p* < 0.05) but higher subcutaneous fat *ACACA* expression (*p* < 0.05) than thin-tailed sheep (Figure 2e,f).

### 3.3. Temporal Expression Patterns of miR-320d and SCD During Preadipocyte Culture and Induction

**Figure 3:**
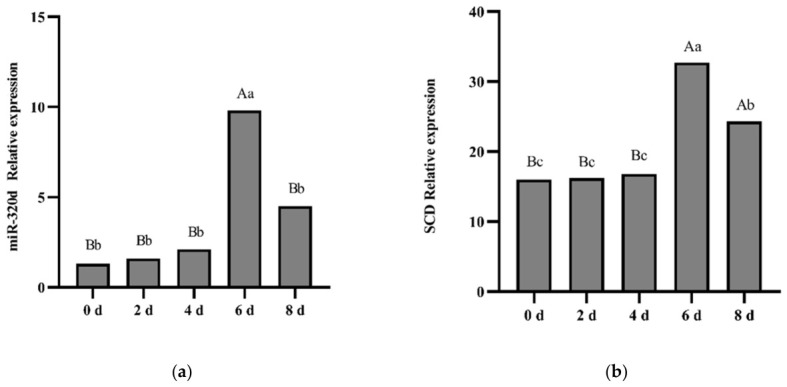
Expression levels of miR-320d and *SCD* during preadipocyte culture and induction. (**a**) Relative expression of miR-320d at different time points (days 0, 2, 4, 6, and 8). (**b**) Relative expression of SCD at different time points (days 0, 2, 4, 6, and 8). Different lowercase and uppercase letters indicate significant differences at *p* < 0.05 and *p* < 0.01, respectively.

miR-320d expression displayed an upward-then-downward trend during the induction of ovine tail preadipocytes (Figure 3), reaching a maximum at day 6 with significantly higher levels than at other time points (*p* < 0.01). *SCD* shared a consistent expression trend with miR-320d, peaking at day 6 with a slight decrease at day 8. Its expression at days 6 and 8 was significantly higher than that at days 0, 2, and 4 (*p* < 0.01), indicating that *SCD* may be highly involved in the development and lipid metabolism of ovine tail preadipocytes.

### 3.4. Validation of the Targeting Relationship Between miR-320d and SCD via Dual-Luciferase Reporter Assay

**Figure 4:**
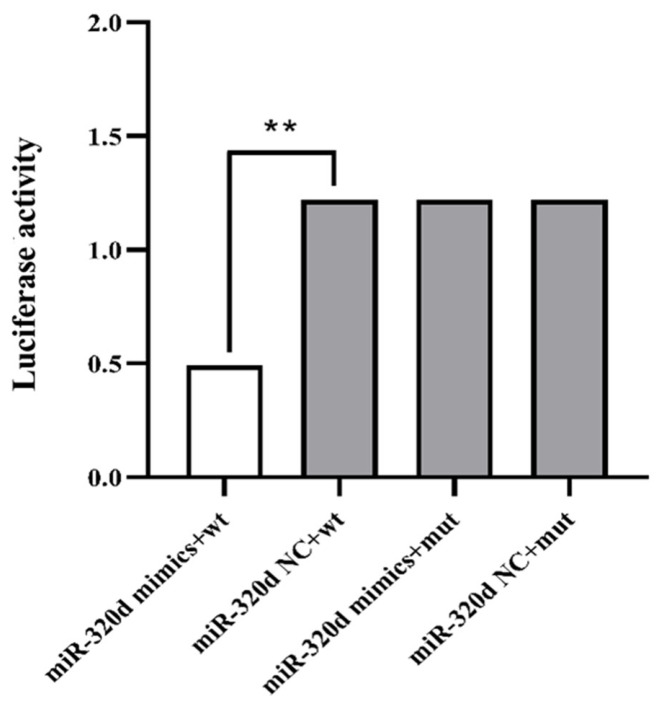
Dual-luciferase reporter assay verifying the targeted binding between miR-320d and SCD. ** Indicates a significant difference at *p* < 0.01.

A dual-luciferase reporter assay was performed to verify the interaction between miR-320d and *SCD*. The miR-320d mimics group showed significantly reduced luciferase activity in the *SCD* wild-type 3′UTR vector (*p* < 0.01) (Figure 4), suggesting that miR-320d specifically binds to the *SCD* 3′UTR to inhibit gene expression. No significant difference in luciferase activity was observed in the mutant-type vector between the mimics and NC groups (*p* > 0.05), further confirming the specific targeted binding of miR-320d to *SCD*.

### 3.5. Regulatory Effects of miR-320d on Cell Proliferation and Fatty Acid Metabolism in Ovine Tail Preadipocytes

**Figure 5:**
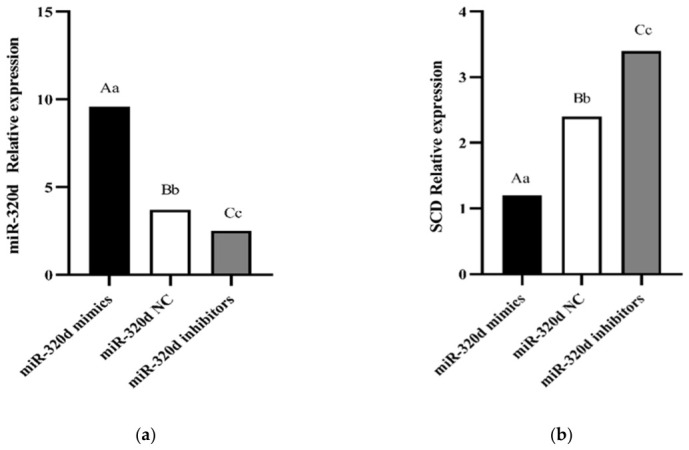
Expression levels of miR-320d and *SCD* post-transfection. (**a**) Relative expression level of miR-320d among different transfection groups. (**b**) Relative expression level of *SCD* among different transfection groups. Different lowercase and uppercase letters indicate significant differences at *p* < 0.05 and *p* < 0.01, respectively.

The levels of miR-320d and *SCD* mRNA were detected at 48 h post-transfection to evaluate transfection efficiency and gene expression changes (Figure 5). The miR-320d overexpression group exhibited significantly elevated miR-320d expression but decreased *SCD* mRNA levels compared with the control and inhibition groups (*p* < 0.01), confirming the inhibitory effect of miR-320d on *SCD* transcription.

**Figure 6:**
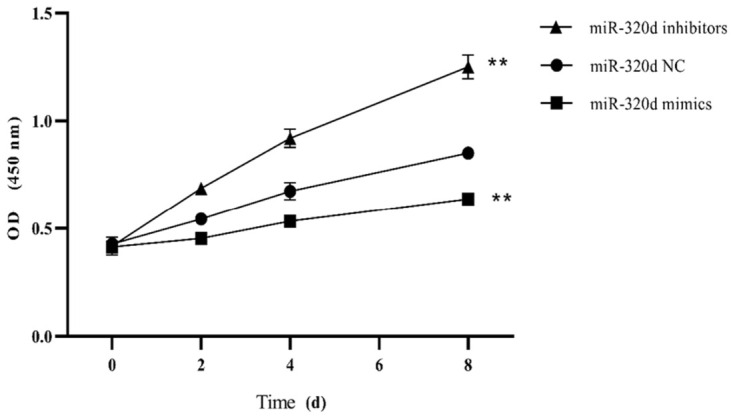
Changes in cell viability among different groups after transfection, measured by CCK-8 assay. ** Indicates a significant difference at *p* < 0.01.

Cell viability assays showed that viability was significantly higher in the miR-320d inhibition group than in the overexpression group (*p* < 0.01) (Figure 6), indicating that miR-320d suppresses the proliferation and viability of ovine tail preadipocytes.

**Figure 7:**
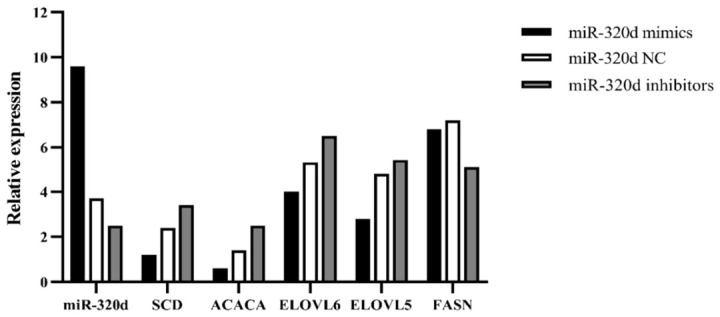
Relative expression levels of miR-320d and functionally related genes in the fatty acid metabolism pathway post-transfection.

Pathway enrichment analysis previously [16] confirmed that *SCD* and its downstream genes (*ACACA*, *ELOVL6*, *ELOVL5*, *FASN*) were enriched in the fatty acid metabolism pathway. Overexpression of miR-320d significantly downregulated these genes (*p* < 0.05), while miR-320d inhibition markedly upregulated their expression (Figure 7). These results demonstrate that *SCD* positively regulates fatty acid metabolism, and miR-320d negatively modulates this pathway by targeting *SCD*, thereby mediating tail fat deposition in sheep.

## 4. Discussion

### 4.1. Rationality of Tissue Sampling and Experimental Design

Fat deposition in sheep is a sophisticated, multi-tissue coordinated biological process, wherein individual tissues exert distinct and irreplaceable roles in lipid synthesis, trafficking, and adipocyte lipid accumulation. Tail fat, a specialized subcutaneous adipose depot in sheep, serves as the primary phenotypic determinant of tail-type variation, with its adipogenic capacity directly shaping fat-tailed and thin-tailed morphological discrepancies [20]. Intramuscular fat content and fatty acid profiles vary considerably across skeletal muscle tissues, which are critical determinants of mutton quality, flavor, and economic value [21]. The liver is the central organ governing systematic de novo lipogenesis and triglyceride synthesis, while the small intestine dominates the absorption and assimilation of lipid metabolites [22]. Furthermore, as typical ruminants, sheep rely on rumen microbial fermentation to convert dietary cellulose into volatile fatty acids, providing major carbon sources for peripheral lipogenesis [1]. In this study, 14 tissue types, including visceral organs, skeletal muscles, and multiple adipose depots, were systematically collected. This comprehensive sampling framework covered core lipid metabolic tissues and non-adipose control tissues, enabling an accurate characterization of the tissue-specific expression patterns of miR-320d, miR-151b, and their downstream target genes (*SCD* and *ACACA*). This multi-tissue profiling strategy effectively clarified the adipose-enriched expression characteristics of candidate molecules, providing solid histological evidence for subsequent functional validation.

### 4.2. Regulatory Mechanism of the miR-320d/SCD Axis in Ovine Tail Fat Deposition

Gene expression analysis revealed that miR-320d was predominantly expressed in the tail adipose tissue of both fat-tailed and thin-tailed sheep genotypes, indicating its potential functional specificity. Notably, adipose miR-320d expression was significantly lower in fat-tailed sheep relative to thin-tailed counterparts, whereas *SCD* expression exhibited an inverse trend. Such opposing expression patterns suggest that miR-320d may negatively modulate tail fat deposition by mediating *SCD* expression in sheep. miR-320d belongs to the conserved miR-320 family, which has been previously implicated in the regulation of insulin sensitivity and energy metabolism [23]. However, its biological function in ruminant lipid metabolism remains largely unreported. *SCD* encodes an endoplasmic reticulum-localized rate-limiting enzyme that catalyzes the synthesis of monounsaturated fatty acids, which is indispensable for lipogenesis and lipid depot expansion [15,24]. Consistent with our findings, previous mammalian studies have demonstrated that *SCD* ablation reduces whole-body fat mass and suppresses hepatic lipogenic signaling [25]. In livestock species, genetic variations of *SCD* are tightly associated with intramuscular fat content and fatty acid composition [26,27,28]. Our dual-luciferase reporter assay verified the direct binding of miR-320d to the 3′UTR of *SCD*, thereby suppressing *SCD* transcription. in vitro functional assays further demonstrated that miR-320d overexpression significantly inhibited the viability and proliferation of ovine tail preadipocytes. At the pathway level, miR-320d negatively regulated the fatty acid metabolic cascade, evidenced by the significant downregulation of *SCD* and its downstream lipogenic genes, including *ACACA*, *ELOVL5*, *ELOVL6*, and *FASN*. These genes are core components of de novo fatty acid synthesis [29,30]. Mechanistically, miR-320d directly targets and represses *SCD* expression, thereby inhibiting the fatty acid metabolic pathway, restricting preadipocyte proliferation, and ultimately reducing tail fat accumulation. Furthermore, the highly divergent expression of the miR-320d/*SCD* axis between fat-tailed and thin-tailed breeds implies its potential role in the evolutionary adaptation and domestication of sheep [31,32]. Our results revealed that miR-320d expression in the tail fat of fat-tailed sheep was significantly downregulated compared to that in thin-tailed sheep, concurrent with an upregulation of *SCD*. We hypothesize that during the long-term artificial selection for energy-storing fat tails, genetic or epigenetic modifications may have naturally suppressed miR-320d transcription in specific breeds. This suppression relieves the inhibitory effect on *SCD*, thereby maintaining a highly active fatty acid synthesis cascade to support excessive tail fat deposition. Thus, the miR-320d/*SCD* module is not merely a metabolic checkpoint, but likely a crucial molecular footprint left by breed divergence.

### 4.3. Potential Regulatory Role of the miR-151b/ACACA Module in Subcutaneous Fat

Adipose tissue exhibits remarkable anatomical and functional heterogeneity, with different fat depots utilizing distinct genetic regulatory networks for expansion and metabolism [33]. In this context, the distinct expression patterns of miR-151b and miR-320d provide striking evidence for the depot-specific regulation of lipid metabolism in sheep. While miR-320d dominates the lipogenic regulation in tail fat, miR-151b exhibits a distinct genotype-dependent high expression restricted to subcutaneous adipose tissues. Adipose miR-151b abundance was significantly lower in fat-tailed sheep than in thin-tailed sheep, whereas its target gene *ACACA* exhibited significantly higher expression in the adipose tissues of fat-tailed sheep. The significant negative correlation between miR-151b and *ACACA* expression implies a potential post-transcriptional regulatory relationship. *ACACA* encodes acetyl-CoA carboxylase, a rate-limiting enzyme that catalyzes the first committed step of fatty acid biosynthesis [29]. It is well established as a key functional gene regulating lipid synthesis and participates in multiple metabolic signaling pathways [30]. To date, limited empirical evidence has linked miR-151b to lipid metabolic regulation in animals. This study is the first to identify the adipose-specific enrichment of miR-151b and its negative correlation with the lipogenic gene *ACACA*. It is speculated that miR-151b may serve as a negative regulator of ovine fat deposition by inhibiting *ACACA*-mediated lipogenesis. However, considering the absence of targeted verification and cellular functional validation in the current study, this regulatory relationship requires further systematic confirmation.

### 4.4. Research Significance and Study Limitations

Adipose tissue expansion is predominantly driven by the proliferation and adipogenic differentiation of preadipocytes [34]. Although the murine 3T3-L1 cell line is widely used in adipogenic research [35], interspecies differences limit its applicability for simulating ruminant-specific lipid metabolism. In this study, primary ovine tail preadipocytes were successfully isolated, providing a species-specific cellular platform. Consistent with in vivo tissue expression results, in vitro assays validated that miR-320d suppresses the proliferation of ovine tail preadipocytes [36]. Nevertheless, several limitations of this study should be acknowledged. First, the direct targeting relationship and biological function of the miR-151b/*ACACA* axis were not experimentally validated, serving only as a preliminary screening result. Second, this study focused on the classical fatty acid metabolic pathway; whether miR-320d regulates tail fat deposition through other signaling networks remains to be elucidated. Third, all mechanistic conclusions regarding miR-320d were derived from in vitro cellular gain- and loss-of-function experiments without specific rescue assays or in vivo phenotypic validation (such as gene knockdown or overexpression in live animals). Future studies will aim to conduct targeted in vivo functional verification to further refine the molecular regulatory network of ovine tail fat deposition. From an applied perspective, the identification of the miR-320d/*SCD* axis offers promising genetic markers for the modern mutton sheep industry. Screening for naturally occurring functional variants (SNPs or InDels) within the precursor sequence of miR-320d or the 3′UTR of *SCD* could be integrated into marker-assisted selection (MAS) or genomic selection breeding programs [37]. Identifying individuals with inherently higher miR-320d activity could accelerate the targeted breeding of novel sheep strains with reduced tail fat mass and higher feed conversion efficiency.

## 5. Conclusions

miR-320d functions as a critical negative regulator of tail fat deposition in sheep. By directly targeting the 3′UTR of *SCD* and suppressing its transcription, this microRNA inhibits the proliferation of tail preadipocytes and downregulates key lipogenic genes (*ACACA*, *ELOVL5*, *ELOVL6*, and *FASN*) in the fatty acid synthesis pathway, thereby limiting excessive fat accumulation in the tail. In parallel, the tissue-specific high expression of miR-151b in subcutaneous adipose tissue and its strong negative correlation with *ACACA* implicate the miR-151b/*ACACA* axis in the regulation of subcutaneous adipogenesis. Collectively, these findings reveal natural post-transcriptional brakes that restrict fat expansion in a depot-specific manner and provide actionable genetic targets for selective breeding programs aimed at reducing tail fat size, improving feed efficiency, and enhancing carcass quality in sheep.

## Figures and Tables

**Table 1:** Primer sequences used for quantitative real-time PCR.

miRNA/Gene	Primer Sequence (5′ to 3′)	Product Length (bp)	Temperature (°C)
U6	F: GTGCTCGCTTCGGCAGCACATAT	106	55
	R: AAAATATGGAACGCTTCACGAA		
*β*-actin	F: CTTCCAGCCTTCCTTCCTGG	180	55
	R: GCCAGGGCAGTGATCTCTTT		
miR-320d	F: GCCGAGAAAGCTGGGTTGAGAG	61	55
	R: CAGTGCAGGGTCCGAGGTAT		
entry 2 miR-151b	F: GCCGAGTCGAGGAGCTCACAGT	65	55
	R: CAGTGCAGGGTCCGAGGTAT		
*SCD*	F: TCGTGCCGTGGTATCTATGG	150	55
	R: GGTTGATGGTCTTGTCGTAAGG		
*ACACA*	F: GCAACCACATCTTCCTCAAC	107	55
	R: CTTCCACAGCCGACTTCC		
*ELOVL6*	F: AATACTGATGAGGTGATGTC	188	55
	R: GGGTGGTGATGAATAAAGG		
*ELOVL5*	F: TCGGACTCACTCTGCTGTCT	136	55
	R: TTCTGACGCTAGGACTGG		
*FASN*	F: GCTTCCTGGTGCTGATGTC	115	55
	R: GTCTGCTTGGCGAACTCC		

## Data Availability

The original contributions presented in the study are included in the article, further inquiries can be directed to the corresponding authors.

## Abbreviations

The following abbreviations are used in this manuscript:

miRNA	microRNA
mRNA	messenger RNA
lncRNA	long non-coding RNA
RT-qPCR	quantitative real-time PCR
3′UTR	3′ untranslated region
WT	wild-type
MUT	mutant-type
NC	negative control
SW	thin-tailed group
ZW	fat-tailed group
CCK-8	Cell Counting Kit-8
PBS	phosphate-buffered saline

## References

[1] Chessa B., Pereira F., Arnaud F., Amorim A., Goyache F., Mainland I., Kao R.R., Pemberton J.M., Beraldi D., Stear M.J. (2009). Revealing the history of sheep domestication using retrovirus integrations. Science.

[2] Wang X., Fang C., He H., Cao H., Liu L., Jiang L., Ma Y., Liu W. (2021). Identification of key genes in sheep fat tail evolution based on rna-seq. Gene.

[3] Su X.-H., He H.-Y., Fang C., Liu L.-L., Liu W.-J. (2023). Transcriptome profiling of lncrnas in sheep tail fat deposition. Anim. Biotechnol..

[4] Mereu P., Pirastru M., Sanna D., Bassu G., Naitana S., Leoni G.G. (2024). Phenotype transition from wild mouflon to domestic sheep. Genet. Sel. Evol..

[5] Wang G., Tian L., Zhang S., He Z., Zhao F., Chang M., Han W., Ye D., Gao J., Li S. (2026). Deciphering the regulatory network of tail fat deposition in large-and small-tailed han sheep through transcriptome and micrornaome profiling. Biology.

[6] Jing X., Zhou J., Degen A., Wang W., Guo Y., Kang J., Liu P., Ding L., Shang Z., Qiu Q. (2020). Comparison between tibetan and small-tailed han sheep in adipocyte phenotype, lipid metabolism and energy homoeostasis regulation of adipose tissues when consuming diets of different energy levels. Br. J. Nutr..

[7] Yardimci M., Hesna Sahin E., Cetingul I., Bayram İ., Altunbas K., Sengor E. (2008). Estimation of carcass composition and fat depots by means of subcutaneous adipocyte area and body and tail measurements in fat-tailed akkaraman lambs. S. Afr. J. Anim. Sci..

[8] Kershaw E.E., Flier J.S. (2004). Adipose tissue as an endocrine organ. J. Clin. Endocrinol. Metab..

[9] Sun K., Kusminski C.M., Scherer P.E. (2011). Adipose tissue remodeling and obesity. J. Clin. Investig..

[10] Xu P., Vernooy S.Y., Guo M., Hay B.A. (2003). The drosophila microrna mir-14 suppresses cell death and is required for normal fat metabolism. Curr. Biol..

[11] Esau C., Kang X., Peralta E., Hanson E., Marcusson E.G., Ravichandran L.V., Sun Y., Koo S., Perera R.J., Jain R. (2004). MicroRNA-143 regulates adipocyte differentiation. J. Biol. Chem..

[12] Han F., Zhou L., Zhao L., Wang L., Liu L., Li H., Qiu J., He J., Liu N. (2021). Identification of miRNA in sheep intramuscular fat and the role of miR-193a-5p in proliferation and differentiation of 3t3-l1. Front. Genet..

[13] Deng K., Ren C., Fan Y., Liu Z., Zhang G., Zhang Y., You P., Wang F. (2020). Mir-27a is an important adipogenesis regulator associated with differential lipid accumulation between intramuscular and subcutaneous adipose tissues of sheep. Domest. Anim. Endocrinol..

[14] Zhou G., Wang X., Yuan C., Kang D., Xu X., Zhou J., Geng R., Yang Y., Yang Z., Chen Y. (2017). Integrating mirna and mrna expression profiling uncovers mirnas underlying fat deposition in sheep. BioMed Res. Int..

[15] Paton C.M., Ntambi J.M. (2009). Biochemical and physiological function of stearoyl-coa desaturase. Am. J. Physiol.-Endocrinol. Metab..

[16] Wang Q., Cao H., Su X., Liu W. (2022). Identification of key mirnas regulating fat metabolism based on rna-seq from fat-tailed sheep and f2 of wild argali. Gene.

[17] Lee S.H., Hossner K.L. (2002). Coordinate regulation of ovine adipose tissue gene expression by propionate. J. Anim. Sci..

[18] Misener S., Krawetz S.A. (2000). Bioinformatics Methods and Protocols.

[19] Schmittgen T.D., Livak K.J. (2008). Analyzing real-time pcr data by the comparative ct method. Nat. Protoc..

[20] Wang W., Pang Z., Zhang S., Yang P., Pan Y., Qiao L., Yang K., Liu J., Wang R., Liu W. (2025). Multi-omics integrated analysis reveals the molecular mechanism of tail fat deposition differences in sheep with different tail types. BMC Genom..

[21] Guo T., Wang X., Zhang Q., Wei L., Liu H., Zhao N., Hu L., Xu S. (2022). Comparative analysis of the composition of fatty acids and metabolites between black tibetan and chaka sheep on the qinghai—Tibet plateau. Animals.

[22] Fei X., Jin M., Yuan Z., Li T., Lu Z., Wang H., Lu J., Quan K., Yang J., He M. (2023). Mirna-seq reveals key micrornas involved in fat metabolism of sheep liver. Front. Genet..

[23] Ling H.Y., Ou H.S., Feng S.D., Zhang X.Y., Tuo Q.H., Chen L.X., Zhu B.Y., Gao Z.P., Tang C.K., Yin W.D. (2009). Changes in microrna (mir) profile and effects of mir-320 in insulin-resistant 3t3-l1 adipocytes. Clin. Exp. Pharmacol. Physiol..

[24] Wang H., Zhong J., Zhang C., Chai Z., Cao H., Wang J., Zhu J., Wang J., Ji Q. (2020). The whole-transcriptome landscape of muscle and adipose tissues reveals the cerna regulation network related to intramuscular fat deposition in yak. BMC Genom..

[25] Guillou H., Zadravec D., Martin P.G., Jacobsson A. (2010). The key roles of elongases and desaturases in mammalian fatty acid metabolism: Insights from transgenic mice. Prog. Lipid Res..

[26] Smith S.B., Kawachi H., Choi C.B., Choi C.W., Wu G., Sawyer J.E. (2009). Cellular regulation of bovine intramuscular adipose tissue development and composition. J. Anim. Sci..

[27] Wu X.X., Yang Z.P., Shi X.K., Li J.Y., Ji D.J., Mao Y.J., Chang L.L., Gao H.J. (2012). Association of scd1 and dgat1 snps with the intramuscular fat traits in chinese simmental cattle and their distribution in eight chinese cattle breeds. Mol. Biol. Rep..

[28] Aali M., Moradi-Shahrbabak H., Moradi-Shahrbabak M., Sadeghi M., Kohram H. (2016). Polymorphism in the scd gene is associated with meat quality and fatty acid composition in iranian fat-and thin-tailed sheep breeds. Livest. Sci..

[29] Mao J., DeMayo F.J., Li H., Abu-Elheiga L., Gu Z., Shaikenov T.E., Kordari P., Chirala S.S., Heird W.C., Wakil S.J. (2006). Liver-specific deletion of acetyl-coa carboxylase 1 reduces hepatic triglyceride accumulation without affecting glucose homeostasis. Proc. Natl. Acad. Sci. USA.

[30] Bakhtiarizadeh M.R., Alamouti A.A. (2020). Rna-seq based genetic variant discovery provides new insights into controlling fat deposition in the tail of sheep. Sci. Rep..

[31] Jiang Y., Xie M., Chen W., Talbot R., Maddox J.F., Faraut T., Wu C., Muzny D.M., Li Y., Zhang W. (2014). The sheep genome illuminates biology of the rumen and lipid metabolism. Science.

[32] Moradi M.H., Nejati-Javaremi A., Moradi-Shahrbabak M., Dodds K.G., McEwan J.C. (2012). Genomic scan of selective sweeps in thin and fat tail sheep breeds for identifying of candidate regions associated with fat deposition. BMC Genet..

[33] Zwick R.K., Guerrero-Juarez C.F., Horsley V., Plikus M.V. (2018). Anatomical, physiological, and functional diversity of adipose tissue. Cell Metab..

[34] Xu Y.-X., Wang B., Jing J.-N., Ma R., Luo Y.-H., Li X., Yan Z., Liu Y.-J., Gao L., Ren Y.-L. (2023). Whole-body adipose tissue multi-omic analyses in sheep reveal molecular mechanisms underlying local adaptation to extreme environments. Commun. Biol..

[35] Otto T.C., Lane M.D. (2005). Adipose development: From stem cell to adipocyte. Crit. Rev. Biochem. Mol. Biol..

[36] Zhang W., Wang S., Yang L., Gao L., Ning C., Xu M., Deng S., Gan S. (2024). Profile of mirnas induced during sheep fat tail development and roles of four key mirnas in proliferation and differentiation of sheep preadipocytes. Front. Vet. Sci..

[37] Moghaddar N., Swan A., Van der Werf J. (2014). Genomic prediction of weight and wool traits in a multi-breed sheep population. Anim. Prod. Sci..

